# P079 A challenging case of Takayasu arteritis presenting with subtle symptoms in a young woman: importance of early recognition and multimodal monitoring

**DOI:** 10.1093/rap/rkag113.109

**Published:** 2026-11-03

**Authors:** Nwe Ni Soe, Caroline Cardy

**Affiliations:** Worcestershire Royal Hospital, Worcestershire, United Kingdom; Worcestershire Royal Hospital, Worcestershire, United Kingdom

## Abstract

**Introduction:**

Large vessel vasculitis (LVV) comprises Takayasu arteritis (TAK) and giant cell arteritis, with TAK predominantly affecting young women. Diagnosis is often delayed because of nonspecific symptoms despite significant vascular inflammation. Early recognition through careful clinical examination, vascular imaging, and prompt immunosuppressive treatment is essential to prevent irreversible vascular complications. We present a case highlighting the diagnostic challenges and evolving management of Takayasu arteritis in a young patient.

**Case description:**

An 18-year-old woman was referred by the vascular team following CT findings suggestive of Takayasu arteritis. She initially presented with a neck lump, and an ultrasound demonstrated bilateral carotid artery wall thickening. Her symptoms were limited to fatigue without constitutional symptoms or limb claudication. During ENT examination, she developed dizziness and syncope on palpation of the carotid arteries. C-reactive protein (CRP) had remained persistently elevated since 2023 (35–133 mg/L), previously attributed to coeliac disease.

Clinical examination revealed reduced bilateral radial, brachial, and carotid pulses, more pronounced on the left, with bruits over the left subclavian and right carotid arteries. Blood pressure measurements demonstrated significant upper- and lower-limb discrepancies. CT aortography confirmed Takayasu arteritis. Treatment with prednisolone 40 mg daily and methotrexate 15 mg weekly was commenced alongside aspirin and apixaban. Four weeks later, CRP fell from 133 to 16 mg/L with resolution of dizziness. Methotrexate was discontinued because of hepatotoxicity and replaced with mycophenolate mofetil. Although carotid Doppler demonstrated improved arterial flow and reduced intima-media thickness, relapse occurred during prednisolone tapering with recurrent inflammatory marker elevation, intermittent arm pain, and cushingoid features. PET-CT demonstrated persistent low-grade vasculitic activity confined to the thoracic aorta. Intravenous tocilizumab was initiated, resulting in normalization of inflammatory markers (CRP <1 mg/L; ESR 2 mm/hour) and sustained clinical improvement.

**Discussion:**

This case illustrates the importance of maintaining a high index of suspicion for Takayasu arteritis in young patients with persistent inflammatory markers and subtle clinical symptoms. Careful vascular examination, including pulse assessment, blood pressure measurements in multiple limbs, and auscultation for bruits, remains fundamental for early diagnosis. Serial imaging and inflammatory markers are complementary tools for monitoring disease activity and treatment response. The case also highlights the need for vigilant monitoring of immunosuppressive toxicity and demonstrates the effectiveness of tocilizumab in achieving remission following relapse during corticosteroid tapering. A multidisciplinary approach involving rheumatology, vascular surgery, and radiology was essential in optimising diagnosis, treatment, and long-term surveillance.

**Key learning points:**

• Takayasu arteritis should be considered in young patients with persistent inflammatory markers and subtle symptoms such as fatigue or dizziness.

• Carotodynia, particularly when associated with raised inflammatory markers, should prompt consideration of Takayasu arteritis and other large vessel vasculitides.

• Blood pressure discrepancies between limbs, diminished peripheral pulses, and arterial bruits are valuable clinical clues to large vessel vasculitis.

• Serial vascular imaging and inflammatory markers are complementary tools for assessing disease activity and monitoring treatment response.

• Immunosuppressive therapy requires careful monitoring for treatment-related adverse effects, including hepatotoxicity and glucocorticoid toxicity.

• Tocilizumab is an effective steroid-sparing treatment option for patients with refractory or relapsing Takayasu arteritis and can achieve sustained clinical and biochemical remission.

